# Prevalence and Severity of Diabetic Gastroparesis Symptoms in Relation to Diabetics in Saudi Arabia

**DOI:** 10.7759/cureus.71920

**Published:** 2024-10-20

**Authors:** Khaled A Yaghmour, Naif A Al Shamrani, Fawaz M Alshamrani, Suliman A Alluhib, Essam A Alghamdi, Waleed S Alghamdi

**Affiliations:** 1 Department of Family Medicine, King Abdulaziz University, Jeddah, SAU; 2 College of Medicine, King Abdulaziz University, Jeddah, SAU; 3 Department of Internal Medicine, Faculty of Medicine, King Abdulaziz University, Jeddah, SAU

**Keywords:** diabetes, gastroparesis, saudi arabia, type 1 diabetes mellitus (t1d), type 2 diabetes mellitus

## Abstract

Background: Gastroparesis is a chronic illness characterized by delayed emptying of the stomach without any mechanical obstruction and therefore presents with conditions such as bloating, early satiety, and abdominal pain, among others. In the Kingdom of Saudi Arabia population, it is a major risk factor because, through review, one can find very limited data on the prevalence and severity of the atrophic gastritis condition among diabetes populations in the region.

Objectives: The objectives of this study were to determine the magnitude and severity of symptoms of gastroparesis in type 1 and type 2 diabetic patients in Saudi Arabia, for information-based decision-making in health practices and policy.

Methods: The design of the study, in the present research, is descriptive, cross-sectional, and non-intervening. Information is directly collected for this study from the 119 diabetic patients, both type 1 and type 2, directly by having interviews and telephone surveys during 2023-2024. Patients belong to the age group of 18-69 years; patient participation in the study is voluntary. The Gastroparesis Cardinal Symptom Index is used for scoring the prevalence followed by the symptoms. Descriptive and inferential statistics with the chi-square test and logistic regression models were used to analyze data.

Results: In this study, 68.4% were type 2 and 63.4% were type 1 diabetic patients and more than half of those with type 2 diabetes were in the age range of 50 to 59 years, while the ages of type 1 diabetic patients were evenly distributed. Key findings from this study are: There was a rate of 22.0% for the occurrence of moderate nausea in type 1 and 29.1% in type 2. Vomiting was infrequent in Type 2 (49.4%) compared to Type 1 (31%). Symptom severity: A significant statistical difference was judged by the feeling of stomach fullness (p = 0.038), with more occurrences among the type 2 DM patients. On univariate analysis, symptoms were more severe in younger patients (< 60 years). There were clear socio-economic disparities; non-Saudi nationals and those of a lower educational and income status reported much more severe symptomatology.

Conclusion: This sign- and symptom-based substantiated research was done in Saudi Arabia to identify whether gastroparesis prevalence and severity differ in type 1 and type 2 diabetic patients. More patients suffering were young and had type 2 diabetes and low socio-economic status. Such findings would surely accentuate the need and call for indispensable, targeted interventions from more longitudinal studies, for a better approach and understanding of diabetic gastroparesis.

## Introduction

Gastroparesis is a chronic disorder where the stomach requires beyond the normal duration to properly empty its contents without any clear blockage [1]. This can lead consequently to a multiplicity of symptoms such as nausea, vomiting, feeling full quickly, bloating, and abdominal pain, all of which can immensely impact a patient's quality of life [2]. The causes are complex in nature and mostly boil down to issues with stomach muscular structure, significant nerve damage, and multiple problems with the digestive system’s nervous network [3].

One of the most significant risk factors for gastroparesis that must be considered is diabetes, for both type 1 and type 2. This is mostly because diabetes can cause continuous damage to the vagus nerve, which holds control of the mobility of food through the digestive tract [4]. Studies have found that approximately 30-50% of patients with longstanding diabetes experience some form of gastroparesis more than others [5].

This issue is specifically worth noticing in places like the Middle East, especially Saudi Arabia, in which diabetes rates are significantly high [6]. Saudi Arabia has been found to have one of the highest rates of diabetes in the globe, affecting more than 25% of adults [7]. Nonetheless, there's not much information available about the commonality or the severity of gastroparesis symptoms among diabetic patients in Saudi Arabia. Such lack of statistical information makes it very difficult to create a properly studied treatment and management plans that suit the well-being of the patients.

The study aims to fill such a gap by excellently examining how common and severe gastroparesis symptoms are among diabetic patients in Saudi Arabia. We have the conviction that gastroparesis is a huge problem in this community and that its manifestations significantly affect their quality of life. By using well-reviewed clinical assessments and validated symptom questionnaires, it was sufficient enough to gather reliable data to guide healthcare practices and policies based upon well-collected data associated with a great level of basic understanding in relation to the topic's knowledge leading to a very interesting discussion in regard many aspects of this topic.

Understanding the effects and outcomes of gastroparesis on diabetic patients in Saudi Arabia is important and worthy of attention for multiple reasons. It will provide very needed help in identifying those at risk, leading to better prevention and treatment strategies and contributing to a much better outcome. It will also indeed contribute to helping healthcare providers in diagnosing and managing the condition more efficiently and effectively and add to the universal understanding of diabetic gastroparesis, especially in places with significant diabetes rates. Ultimately, this research aims to positively enhance patient general state of health and improve the currently followed methods of diabetes care in Saudi Arabia. In summary, this study seeks to provide the needed data on the prevalence and severity of gastroparesis symptoms among diabetic patients in Saudi Arabia. The different insights gained throughout the research will surely help in establishing better management and intervention strategies, leading to enhanced health and quality of life for those affected by such abnormality.

## Materials and methods

Study design

A descriptive cross-sectional non-interventional design is used to explain the prevalence and severity of diabetic gastroparesis in patients diagnosed with type 1 and type 2 diabetes in the Kingdom of Saudi Arabia. The research will be conducted over the period of 2023 to 2024 by using direct interviews as well as phone call-based surveys to ensure that a maximum amount of data is compiled from diverse demographic and geographic strata.

Population

The sample for the study comprises diabetic subjects residing in Saudi Arabia, who can be further classified into type 1 and type 2 diabetes. The study population comprises participants selected in the age group of 18 to 69 years, thus making a wide variation among the adult diabetic population. In a highly systematic manner, all the participants with the experience of pre-diabetic gastroparesis symptom history were excluded, to analyze only fresh cases.

Sampling

An expected sample size of 383 participants was estimated in such a way that the statistical test of power will be competent; unfortunately, due to many limitations in the quality of the data and methods of collection, it has been only possible to collect data of 119 participants. The sample was recruited on a voluntary basis. It will help them self-select themselves for the study and help us to get a dataset that is diverse and representative in nature.

Data collection

Data was collected using a well-prepared questionnaire based upon the contents of the Gastroparesis Cardinal Symptom Index (GCSI) which is a validated tool used to measure the severity of gastroparesis symptoms (Table 1). It assigns a score from 0 to 5 for nine symptoms, such as nausea, vomiting, bloating, and loss of appetite, based on the patient’s perception. Such a tool was administered over the telephone and through direct interviews. This will ensure the capture of large volumes of symptomatic, demographic, and clinical data relevant to diabetic gastroparesis. Descriptive statistics were used in surveying the demographic environment and in establishing the prevalence of symptoms. Inferential statistics are used through chi-square testing of the independent samples in relation to categorical variables in addition to logistic regression models, and the relationship of symptoms with gastroparesis and diabetic types is explored. Significance is set at p<0.05, with 95% confidence interval.

**Table 1:** GCSI questionnaire used to collect the data during the interviews GCSI: Gastroparesis Cardinal Symptom Index

Symptom	None	Very Mild	Mild	Moderate	Severe	Very Severe
Nausea (feeling sick to your stomach as if you were going to vomit)	0	1	2	3	4	5
Retching (heaving as if to vomit but nothing comes up)	0	1	2	3	4	5
Vomiting	0	1	2	3	4	5
Stomach fullness	0	1	2	3	4	5
Not able to finish a normal-sized meal	0	1	2	3	4	5
Feeling excessively full after meals	0	1	2	3	4	5
Loss of appetite	0	1	2	3	4	5
Bloating (feeling like you need to loosen your clothes)	0	1	2	3	4	5
Stomach or belly visibly larger	0	1	2	3	4	5

Data analysis

The quantitative data were processed using IBM SPSS Statistics for Windows, Version 21 (Released 2012; IBM Corp., Armonk, New York, United States). Descriptive statistics were used for the purpose of illustrating the demographic landscape and the prevalence of symptoms. Also in the study are inferential statistics on the associations between symptoms of gastroparesis with diabetes types and between-group differences, through the chi-square test for categorical variables and logistic regression models. The threshold for significance was p < 0.05 with a confidence level of 95%.

Ethical considerations

There is complete ethical compliance to be adhered to in which all the participants must give their informed consent before being involved in the study. Information about the participants is very confidential as there are measures to ensure that data anonymization will not lead to leaking personal information. The study attains ethical approval from the relevant institutional review board (IRB #553-23) of King Abdulaziz University Hospital.

Limitations and bias mitigation

Volunteer sampling and self-reported data are possible biases that have been fully accepted in this study; therefore, careful questionnaire design and rigorous methods of data validation are to be put in place. Information and recall biases are managed through methodological rigor and self-reported data that are validated by medical records where possible. The study has several limitations that should be acknowledged. First is the use of the Gastroparesis Cardinal Symptom Index (GCSI) questionnaire which relies on patient self-reporting and introduces potential recall and response biases as it has been mentioned, as patients may underreport or overestimate their symptoms based on their perception or recollection. Additionally, since the GCSI does not include objective assessments such as gastric emptying studies, there is a possibility of misclassification or overestimation of gastroparesis prevalence. Another limitation arises from the cross-sectional nature of the study, which precludes establishing causality or understanding changes in symptom severity over time. Furthermore, the study may face challenges in generalizing the findings, as the sample might not be fully representative of the entire diabetic population in Saudi Arabia due to geographical, cultural, or healthcare accessibility variations.

## Results

The age of the majority of type 2 diabetes patients had been spread over two groups: 50-59 years (44.3%) and 60-69 years (35.4%) (Table 2). The type 1 group wasn’t skewed to any particular group and was uniformly distributed across the five ranges. The gender distribution for males is around 60% and females around 40%. The majority of participants are Saudis, making up 68.4% for type 2 and 63.4% for type 1, and 83.5% of type 2 patients were married compared to 70.7% of type 1 patients. Most participants with type 1 had high school as their highest level of education (29.3%), with a bachelor’s degree as the second highest 24.4%, while the highest education degree in the type 2 group was a bachelor’s degree (22.8%) and around 45% was similarly distributed among high, middle, and primary school degrees. Many patients were unemployed: 36.7% for type 2 and 41.5% for type 1, with many earning less than 5000 SAR per month. The majority of participants came from the Western Region (97.5% for type 2 and 95.1% for type 1).

**Table 2:** Demographic characteristics of patients who participated in the questionnaire

Variable		Diabetes Type			
		Type 1 Diabetes		Type 2 Diabetes	
		N	%	N	%
Age	18 - 29	8	19.5%	1	1.3%
	30 - 39	7	17.1%	4	5.1%
	40 - 49	8	19.5%	11	13.9%
	50 - 59	9	22.0%	35	44.3%
	60 - 69	9	22.0%	28	35.4%
Gender	Female	18	43.9%	32	40.5%
	Male	23	56.1%	47	59.5%
Nationality	Non-Saudi	15	36.6%	25	31.6%
	Saudi	26	63.4%	54	68.4%
Marital Status	Divorced	1	2.4%	1	1.3%
	Married	29	70.7%	66	83.5%
	Single	11	26.8%	4	5.1%
	Widow	0	0.0%	8	10.1%
Educational Level	Higher Education	1	2.4%	6	7.6%
	Bachelor’s degree	10	24.4%	18	22.8%
	Diploma	4	9.8%	3	3.8%
	High school	12	29.3%	15	19.0%
	Middle school	6	14.6%	17	21.5%
	Primary school	3	7.3%	13	16.5%
	Non-educated	5	12.2%	7	8.9%
Occupation	Craft labor	3	7.3%	3	3.8%
	Education sector	3	7.3%	7	8.9%
	Freelancer	2	4.9%	4	5.1%
	Healthcare provider	1	2.4%	1	1.3%
	military	2	4.9%	2	2.5%
	Office work	6	14.6%	13	16.5%
	Retired	6	14.6%	20	25.3%
	Student	1	2.4%	0	0.0%
	Unemployed	17	41.5%	29	36.7%
Estimated Monthly Income	<5000	23	56.1%	36	45.6%
	5000-10000	14	34.1%	31	39.2%
	10000-15000	3	7.3%	9	11.4%
	15000-20000	1	2.4%	3	3.8%
Residency	Central Region	0	0.0%	1	1.3%
	Southern Region	2	4.9%	1	1.3%
	Western Region	39	95.1%	77	97.5%

Table 3 shows the prevalence and severity of gastroparesis symptoms among the participants. Nausea, vomiting, and retching showed no significant differences between the two groups: types 1 and 2 DM patients. Stomach fullness had a high significance with a P-value of 0.038. The rest of the symptoms had no high significant value.

**Table 3:** Correlational chi-square analysis between different gastroparesis complaints among diabetic patients

Symptom	Severity	Type 1 Diabetes N	Type 1 Diabetes %	Type 2 Diabetes N	Type 2 Diabetes %	P-value
Nausea	None	0	0.0%	39	49.4%	0.8
Nausea	Very mild	8	19.5%	16	20.3%	0.8
Nausea	Mild	6	14.6%	13	16.5%	0.8
Nausea	Moderate	7	17.1%	8	10.1%	0.8
Nausea	Severe	9	22.0%	23	29.1%	0.8
Nausea	Very severe	2	4.9%	2	2.5%	0.8
Vomiting	None	13	31.7%	41	49.4%	0.1
Vomiting	Very mild	11	26.8%	23	29.1%	0.1
Vomiting	Mild	7	17.1%	13	16.5%	0.1
Vomiting	Moderate	3	7.3%	5	6.3%	0.1
Vomiting	Severe	0	0.0%	2	2.5%	0.1
Vomiting	Very severe	3	7.3%	2	2.5%	0.1
Retching	None	17	41.5%	35	44.3%	0.997
Retching	Very mild	12	29.3%	21	26.6%	0.997
Retching	Mild	12	29.3%	18	22.8%	0.997
Retching	Moderate	10	24.4%	23	29.1%	0.997
Retching	Severe	0	0.0%	2	2.5%	0.997
Retching	Very severe	3	7.3%	2	2.5%	0.997
Stomach Fullness	None	12	29.3%	38	48.1%	0.038
Stomach Fullness	Very mild	1	2.4%	19	24.1%	0.038
Stomach Fullness	Mild	4	9.8%	17	21.5%	0.038
Stomach Fullness	Moderate	13	31.7%	22	27.8%	0.038
Stomach Fullness	Severe	1	2.4%	2	2.5%	0.038
Stomach Fullness	Very severe	1	2.4%	3	3.8%	0.038
Not being able to finish a normal-sized meal	None	10	24.4%	17	21.5%	0.122
Not being able to finish a normal-sized meal	Very mild	4	9.8%	12	15.2%	0.122
Not being able to finish a normal-sized meal	Mild	5	12.2%	14	17.7%	0.122
Not being able to finish a normal-sized meal	Moderate	9	22.0%	20	25.3%	0.122
Not being able to finish a normal-sized meal	Severe	5	12.2%	6	7.6%	0.122
Not being able to finish a normal-sized meal	Very severe	1	2.4%	1	1.3%	0.122
Feeling excessively full after meals	None	12	29.3%	13	16.5%	0.392
Feeling excessively full after meals	Very mild	5	12.2%	18	22.8%	0.392
Feeling excessively full after meals	Mild	7	17.1%	19	24.1%	0.392
Feeling excessively full after meals	Moderate	11	26.8%	20	25.3%	0.392
Feeling excessively full after meals	Severe	7	17.1%	11	13.9%	0.392
Feeling excessively full after meals	Very severe	3	7.3%	0	0.0%	0.392
Loss of appetite	None	10	24.4%	20	25.3%	0.293
Loss of appetite	Very mild	5	12.2%	18	22.8%	0.293
Loss of appetite	Mild	7	17.1%	19	24.1%	0.293
Loss of appetite	Moderate	11	26.8%	20	25.3%	0.293
Loss of appetite	Severe	7	17.1%	11	13.9%	0.293
Loss of appetite	Very severe	3	7.3%	3	3.8%	0.293
Bloating	None	7	17.1%	14	17.7%	0.974
Bloating	Very mild	9	22.0%	14	17.7%	0.974
Bloating	Mild	7	17.1%	13	16.5%	0.974
Bloating	Moderate	8	19.5%	17	21.5%	0.974
Bloating	Severe	7	17.1%	11	13.9%	0.974
Bloating	Very severe	3	7.3%	3	3.8%	0.974
Stomach Size	None	13	31.7%	24	30.4%	0.847
Stomach Size	Very mild	7	17.1%	19	24.1%	0.847
Stomach Size	Mild	7	17.1%	19	24.1%	0.847
Stomach Size	Moderate	4	9.8%	4	5.1%	0.847
Stomach Size	Severe	2	4.9%	2	2.5%	0.847
Stomach Size	Very severe	4	9.8%	4	5.1%	0.847

Table 4 shows the different biological variables among participants. The average participant's weight is 79 ± 16.2 kg, the height is 167.6 ± 8.8 cm, and BMI is 28 ± 5.5 kg/m². The mean HbA1c level was 8.11% with a standard deviation of 1.97%.

**Table 4:** Descriptive statistics for different quantitative biological variables among participants

Variable	N	Minimum	Maximum	Mean	Std. Deviation	Variance
Weight	119	45	130	79.00	16.263	264.494
Height	119	145.0	186.0	167.658	8.8465	78.260
BMI	119	17.72	47.75	28.0608	5.55410	30.848
HbA1C	119	6	17	8.11	1.973	3.892

**Figure 1:**
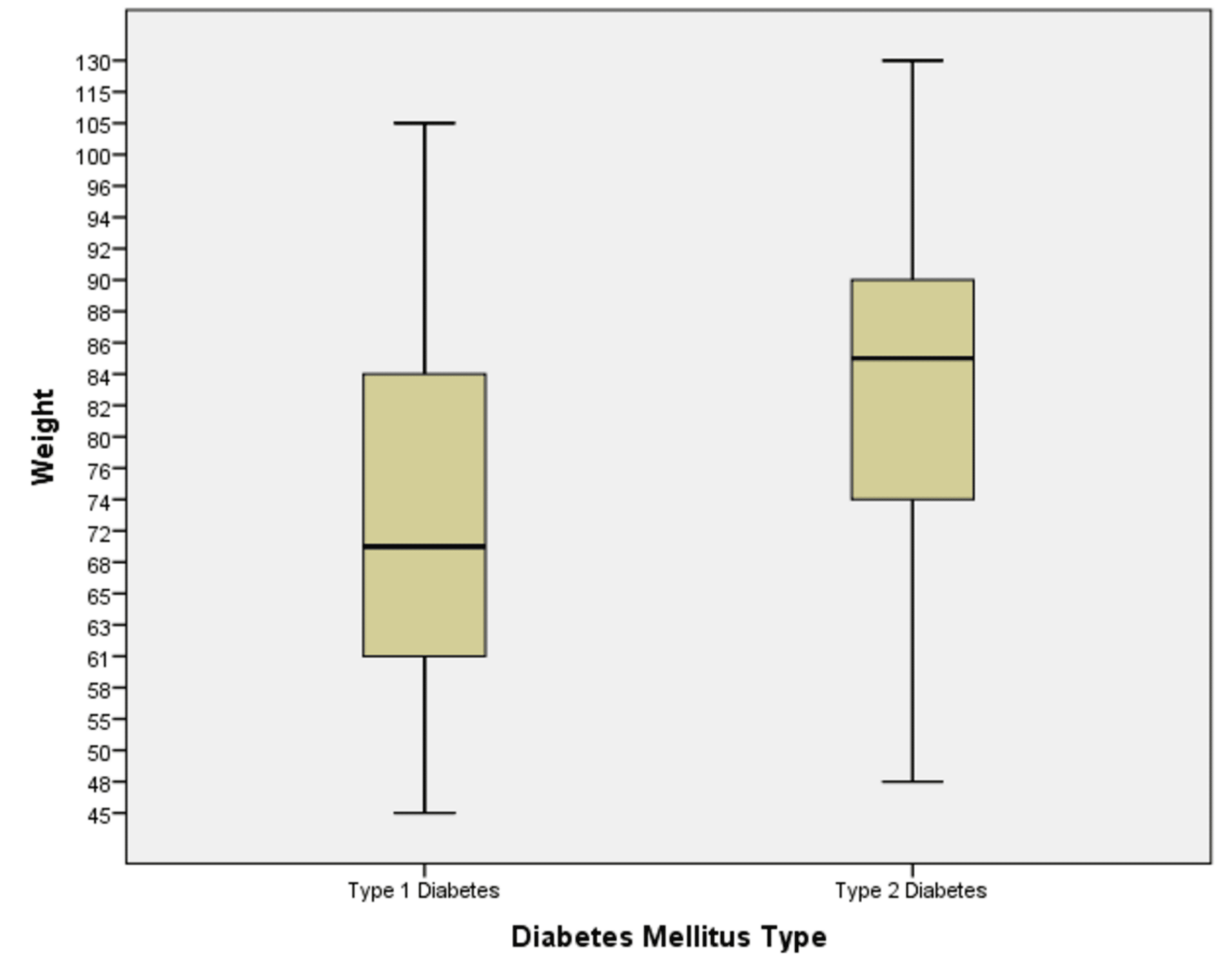
Weight distribution among different diabetic patients

Figure 1 shows the weight distribution among the diabetic patients using a boxplot. The boxplot displays the median weight, the spread of the middle 50% of the data (interquartile range), and any outliers. This visual summary highlights the variability in weight among the participants, showing the range and any extreme values in the dataset.

**Figure 2:**
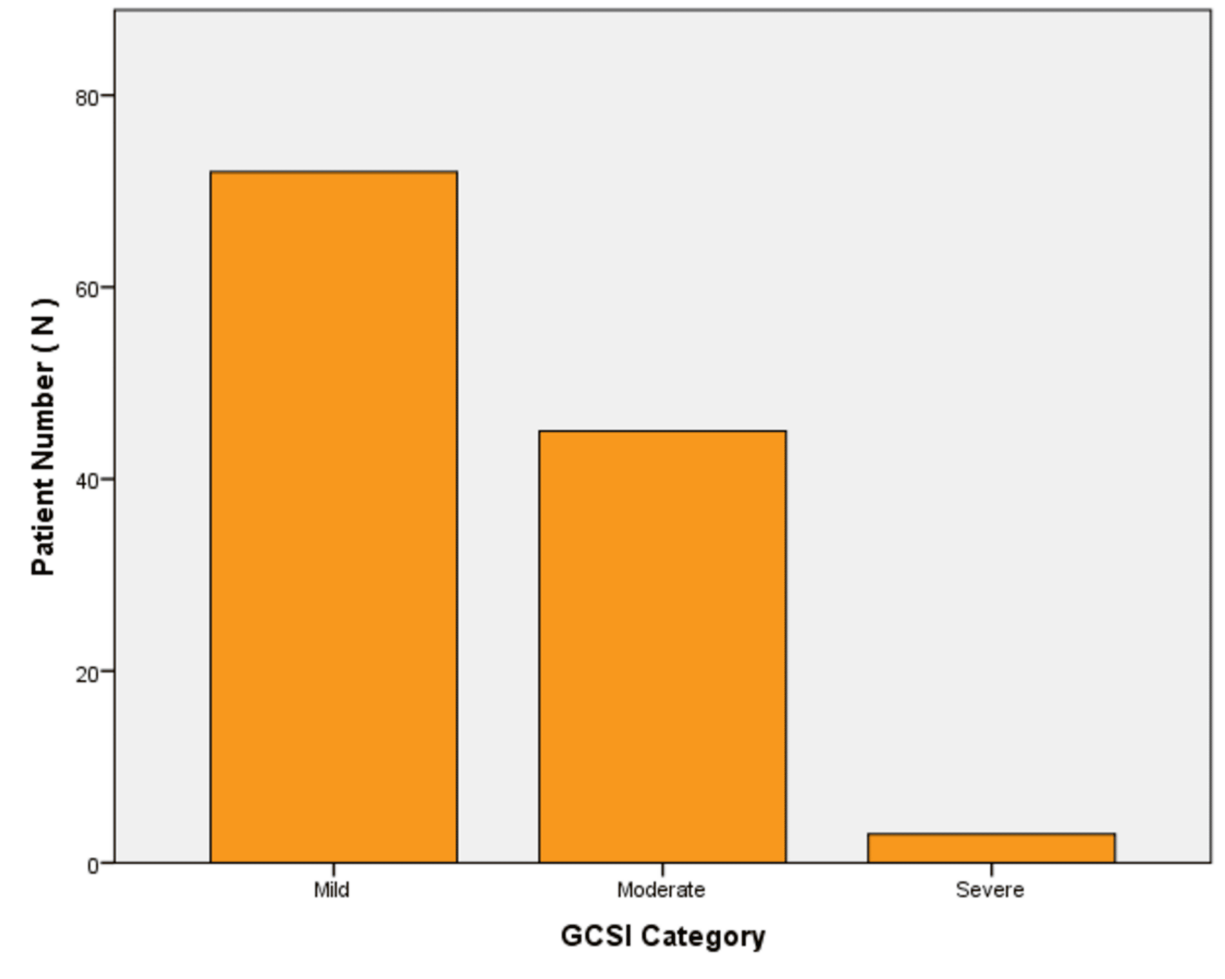
Distribution of patients by the GCSI category GCSI: Gastroparesis Cardinal Symptom Index

Patients were grouped according to their GCSI scores as mild, moderate, or severe. Figure 2 illustrates how patients are distributed across these categories. Most patients were classified as having mild symptoms, while the moderate category had the next second-highest number of patients, with fewer falling into the severe category.

**Figure 3:**
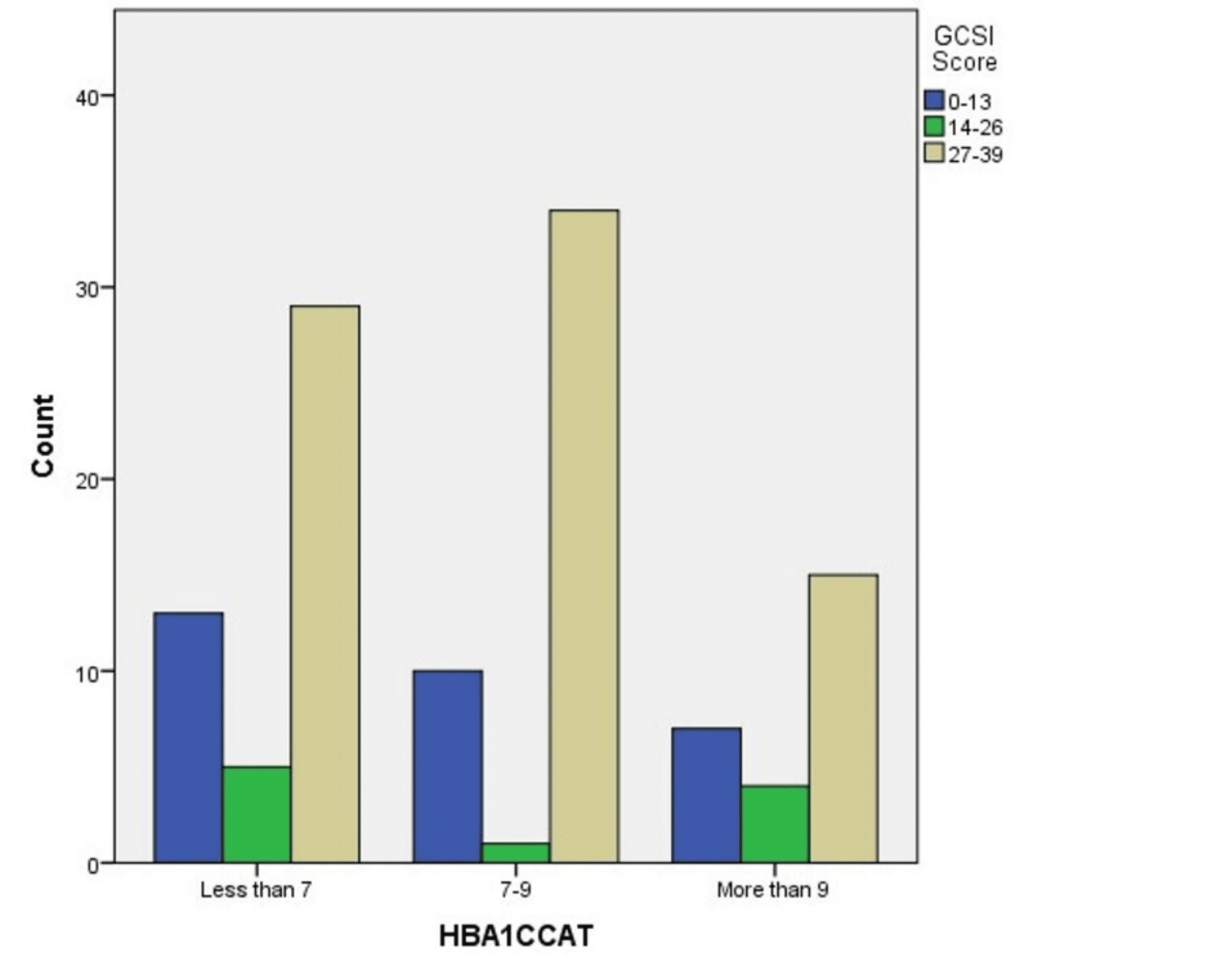
Distribution of GCSI scores based on HbA1c categories GCSI: Gastroparesis Cardinal Symptom Index

HbA1c levels were divided into three categories as follows: controlled (<7%), high (7-9%), and uncontrolled (>9%) HbA1c levels. As shown in Figure 3, the highest prevalence for both type 1 & 2 diabetes was in group (7-9%) followed by the controlled and lastly the uncontrolled group. Figure 3 illustrates the distribution of GCSI scores across different HbA1c categories, classified as "Less than 7", "7-9", and "More than 9". The GCSI scores are grouped into three categories: low (0-13), moderate (14-26), and high (27-39). The data shows that the highest frequency of severe GCSI scores (27-39) is observed in patients with HbA1c levels between "7-9".

## Discussion

In this study, the symptoms of gastroparesis were assessed among diabetics in Saudi Arabia with the aim of revealing valuable information regarding the spectrum of such symptoms with the most predominant in both type 1 and type 2 diabetes mellitus. The only limitation faced in this study is the reduction of the sample size from 383 participants as it was estimated initially in such a way that the statistical test of power would be competent; unfortunately due to many limitations in the quality of the data and methods of collection, it has been only possible to collect 119 participants in total. Nonetheless, still, it can be deduced from this data that a larger number of diabetic patients experienced moderate to severe symptoms of gastroparesis, with minor variations between the two types of diabetes.

Prevalence of symptoms

From this study, the most reported symptoms by the subjects were moderate stomach fullness, moderate nausea, and moderate to acute inability to finish a normal-sized meal. From the data, 27.8% of the patients with type 2 diabetes achieved moderate stomach fullness while 31.7% of patients with type 1 diabetes underwent a similar symptom. These findings are in coincidence with the reports of Almogbel et al., where bloating was identified in 63.94%, stomach fullness in 55.1%, and early satiety in 48.3% of the cases [8]. So, stomach fullness and bloating are reported as the major symptoms in both this study and those of others.

The reported another prevailing symptom was that of nausea with the same finding in 22.0% of the patients with type 1 diabetes and 29.1% of the type 2 diabetes patients. These have come to coincide with observations of Asghar et al., where early satiety was detected in 45.1%, stomach fullness in 44.5%, and nausea in 33.1% of the general population of diabetic patients [9]. The symptoms that coincide with other studies and the current one place significant importance on the characteristics of the symptoms described as contributors to the clinical manifestations of gastroparesis among diabetic patients.

Vomiting symptoms were less reported with 49.4% of the type 2 diabetes patients and 31.7% of the type 1 diabetes patients indicating no vomiting. These findings are in line with Almogbel et al., who also found vomiting to be one of the least common presenting symptoms in their cohort [8]. The lesser prevalence of vomiting as compared to other symptoms, a sign of which could be important, indicates that the symptom may not be as widespread as nausea, bloating, or stomach fullness in diabetic gastroparesis.

Inability to finish a normal-sized meal was seen in 25.3% of patients with type 2 diabetes and 22.0% of those with type 1 diabetes. The symptom, along with the feeling of excessive fullness after eating meals, indicates the delay in gastric emptying, which is the hallmark of gastroparesis. These results are in agreement with those of Asghar et al., who also found that early satiety and postprandial fullness were common symptoms in their participants [9].

Intensity of symptoms

The analysis of the seriousness of these symptoms in diabetic patients uncovered some crucial findings; 22.0% of those with type 1 diabetes, while 21.5% of those with type 2 diabetes had gross severe nausea with discomfort. The degree of nausea one has can adversely affect the quantity of food eaten hence leading to malnutrition. In this study, 12.2% of those with type 1 diabetes, whereas 7.6% of those with type 2 diabetes had gross severe stomach fullness and reported severe bloating. The severe symptoms caused noticeable physical pain and mental suffering, leading to being socially isolated and a lower quality of life. Moreover, 22.0% of individuals with type 1 diabetes and 25.3% of those with type 2 diabetes stated that they could not finish a standard meal. This symbol of delayed digestion interrupts regular eating patterns, resulting in weight loss and malnutrition. Although not common, vomiting posed a significant health risk for 7.3% of type 1 diabetes patients reporting moderate severity, and 6.3% of type 2 diabetes patients, while 2.5% of type 2 experienced severe vomiting. Excessive vomiting can result in dehydration and disruptions in electrolyte levels, requiring medical intervention. These results support the research by Gourcerol et al., showing a connection between delayed gastric emptying and higher mortality rates in people with diabetes [10]. This highlights the pressing requirement for prompt and efficient management of severe gastroparesis symptoms to enhance patient outcomes. Furthermore, Parkman et al. emphasized that not only the speed of gastric emptying but also issues like impaired gastric accommodation affect the intensity of gastroparesis symptoms [11]. This implies that managing the severity of gastroparesis symptoms effectively requires a comprehensive treatment approach that considers both gastric motility and other underlying factors.

Correlation between HbA1c and GCSI scores

The distribution of GCSI scores across different HbA1c categories in Figure 4 highlights a notable and complex relationship between glycemic control and gastrointestinal symptom severity among diabetic patients. Interestingly, the highest frequency of severe gastrointestinal symptoms, represented by GCSI scores of 27-39, is observed in patients with HbA1c levels ranging between 7 and 9. This finding suggests that individuals with intermediate glycemic control experience more severe gastroparesis symptoms compared to those with HbA1c levels either lower than 7 or greater than 9. This observation contrasts with the common expectation that higher HbA1c levels, indicative of poorer glycemic control, would correlate with increased severity of gastrointestinal symptoms. One possible explanation for the higher symptom burden in the intermediate HbA1c group is that these patients may have fluctuating blood glucose levels, which can exacerbate autonomic dysfunction and lead to more pronounced gastroparesis symptoms. Additionally, patients within this HbA1c range may have been undergoing more aggressive diabetes management strategies, such as insulin adjustments or the use of GLP-1 agonists, which are known to cause gastrointestinal side effects like nausea and delayed gastric emptying. Conversely, the "More than 9" HbA1c category, representing those with the poorest glycemic control, shows a lower frequency of severe symptoms. This could be due to several factors. Patients with higher HbA1c levels may have developed a certain degree of tolerance to gastrointestinal symptoms over time, leading to underreporting or a lower perception of symptom severity. Another possibility is that these patients could be receiving targeted treatments for their gastrointestinal symptoms, which might attenuate the severity of their condition, thus skewing the results toward lower GCSI scores. Furthermore, the "Less than 7" category, which reflects patients with good glycemic control, shows a relatively lower frequency of severe GCSI scores. This aligns with the traditional view that better glycemic control is associated with fewer complications, including gastrointestinal issues. However, the presence of some patients with severe symptoms in this group indicates that good glycemic control does not fully eliminate the risk of gastroparesis, emphasizing the multifactorial nature of this condition.

Comparison of the study findings to previous studies

In comparison between this study's findings to other similar studies, it has been found that the symptoms of gastroparesis in diabetic patients are the same in two articles, by Almogbel et al. Saudi Arabia [8] and Asghar et al. Pakistan [9], found similar symptoms which means these symptoms happen a lot in people with diabetes. This shows how important it is to see and treat these symptoms to make life better for diabetic people. Studies show that gastroparesis symptoms are linked to bad control of blood sugar and having diabetes for a long time. Almogbel et al. found that symptoms were linked to HbA1c (p=0.001), blood sugar levels (p=0.003), and how long someone had diabetes (p=0.02) [8]. Asghar et al. also saw that symptoms were linked to having diabetes for more than 10 years, high HbA1c (p=0.001), and high fasting blood sugar (p=0.003) [9]. This shows how important it is to control blood sugar well to manage gastroparesis symptoms. Also, Sogabe et al. showed that getting good control of blood sugar helps with stomach movement and eases upper stomach symptoms in diabetic people with gastroparesis, which shows how important it is to control blood sugar [12].

## Conclusions

The study aimed to evaluate the severity and prevalence of symptoms associated with gastroparesis among diabetic patients in Saudi Arabia. It has been found that the most prevalent symptoms are moderate stomach fullness (27.8% in type 2, 31.7% in type 1), nausea (29.1% in type 2, 22.0% in type 1), and inability to finish a meal (25.3% in type 2, 22.0% in type 1) align with previous studies, highlighting stomach fullness, bloating, and delayed gastric emptying as key features of diabetic gastroparesis, with vomiting being less common. Targeted interventions are essential, given the significantly higher prevalence rates among diabetic patients, particularly younger individuals diagnosed with type 2 diabetes. Effective management of diabetic gastroparesis involves strict blood sugar control, customized dietary plans, medications to enhance gastric motility, and treatments for nausea and dehydration.

## References

[1] Camilleri M, Parkman HP, Shafi MA, Abell TL, Gerson L (2013). Clinical guideline: management of gastroparesis. Am J Gastroenterol.

[2] Parkman HP, Yates K, Hasler WL (2011). Clinical features of idiopathic gastroparesis vary with sex, body mass, symptom onset, delay in gastric emptying, and gastroparesis severity. Gastroenterology.

[3] Sarnelli G, Caenepeel P, Geypens B, Janssens J, Tack J (2003). Symptoms associated with impaired gastric emptying of solids and liquids in functional dyspepsia. Am J Gastroenterol.

[4] Bharucha AE, Kudva YC, Prichard DO (2019). Diabetic gastroparesis. Endocr Rev.

[5] Jones MP, Maganti K (2003). A systematic review of surgical therapy for gastroparesis. Am J Gastroenterol.

[6] Alhowaish AK (2013). Economic costs of diabetes in Saudi Arabia. J Family Community Med.

[7] Alqurashi KA, Aljabri KS, Bokhari SA (2011). Prevalence of diabetes mellitus in a Saudi community. Ann Saudi Med.

[8] Almogbel RA, Alhussan FA, Alnasser SA, Algeffari MA (2016). Prevalence and risk factors of gastroparesis-related symptoms among patients with type 2 diabetes. Int J Health Sci (Qassim).

[9] Asghar S, Asghar S, Shahid S, Sajjad H, Abdul Nasir J, Usman M (2023). Gastroparesis-related symptoms in patients with type 2 diabetes mellitus: early detection, risk factors, and prevalence. Cureus.

[10] Gourcerol G, Melchior C, Wuestenberghs F (2022). Delayed gastric emptying as an independent predictor of mortality in gastroparesis. Aliment Pharmacol Ther.

[11] Parkman HP, Natta ML, Maurer AH (2022). Postprandial symptoms in patients with symptoms of gastroparesis: roles of gastric emptying and accommodation. Am J Physiol.

[12] Sogabe M, Okahisa T, Tsujigami K (2005). Ultrasonographic assessment of gastric motility in diabetic gastroparesis before and after attaining glycemic control. J Gastroenterol.

